# Development of a novel SNP assay to detect lactase persistence associated genetic variants

**DOI:** 10.1007/s11033-021-06698-y

**Published:** 2021-09-13

**Authors:** Pasquale De Luca, Daniela Iaconis, Elio Biffali, Coluccia Enza, Laura de Magistris, Gabriele Riegler, Diego Pappalardo, Maria Rosaria Amato, Patrizia Iardino, Concetta Montanino, Bruna De Felice

**Affiliations:** 1 Department of Research Infrastructures for Marine Biological Resources (RIMAR), Sequencing and Molecular Analyses Center, Stazione Zoologica Anton Dohrn, Villa Comunale, 80121 Naples, Italy; 2 Department of Precision Medicine, University of Campania Luigi Vanvitelli, 81100 Caserta, Italy; 3 UOC Clinic and Molecular Pathology, University of Campania Luigi Vanvitelli, 81100 Caserta, Italy; 4 Department of Environmental, Biological and Pharmaceutical Sciences and Technologies (DISTABIF), University of Campania Luigi Vanvitelli, Via Vivaldi 43, 81100 Caserta, Italy

**Keywords:** SNP, Lactase, Fingerprint, Genomic

## Abstract

**Background:**

In adulthood the activity of the lactase enzyme is inherited as autosomal dominant form associated to Single nucleotide polymorphisms (SNPs). The present research was aimed to develop a novel genetic method to test lactase non persistence more powerfully.

**Methods and results:**

In our study, we selected eight different SNPs that are associated with lactase persistence from Caucasian, Arabian Bedouins, sub-Saharian Africans and Asian populations to set up an approach to detect all the eight different SNPs at the same time in the same sample. This technique is centred on the identification of SNPs with a single nucleotide primer extension method using Sanger sequencing and capillary electrophoresis.

**Conclusions:**

Our method allowed us to check the genotype asset of eight SNPs related to lactase persistence simultaneously and in a very efficient manner. It could be applied to a higher number of SNPs in a single reaction.

## Introduction

The most common worldwide food adverse reaction is Lactose intolerance. It is the consequence of the lack or decreased activity of the enzyme lactase, expressed as a member of the brush border on top of microvilli in the small intestine. After the intake of dairy food, individuals bearing such a condition show clinical symptoms such as cramping, abdominal pain, flatulence, diarrhoea, nausea and vomiting. Symptoms are the consequence of bacterial fermentation in large intestine, where free lactose becomes a substrate metabolised into lactic acid, methane, hydrogen and other products [1, 2].

Primary lactose intolerance (adult acquired hypolactasia) is a natural process, involving a gradual disappearance of the activity of Lactase-phlorizin hydrolase (LPH) in the small intestinal brush border, starting between the age of 1–5 years, progressively declining after weaning [3], and depending on the ethnic group. Such phenomenon is very frequent among Asians, in the Mediterranean, and among black individuals, in 60% to 100% of the adult population.

Persistence (LP) or non-persistence (LNP) of lactase expression into adult life is a polymorphic trait that has been initially attributed to the single nucleotide polymorphism C/T–13910 (rs4988235) in an enhancer element 13.9 kb upstream of the lactase gene on chromosome 2 [4], specifically in a sequence known to be target for the transcription factor Oct-1. The − 13910*T allele occurs at very high frequency in northern Europeans and increases the affinity of the surrounding sequence for Oct-1 [5], providing a possible mechanism for up-regulation of *LCT* expression in lactase persistence. It is located in intron 13 of an adjacent gene, *MCM6* (minichromosome maintenance 6). Later, other SNPs have been identified within the same enhancer region. Two of them, the abovementioned C/T –13910 (rs4988235) and G/A –22018 (rs182549), showed complete co-segregation with LNP/LP trait in Europeans [4]. The relation between the C/T –13910 and G/A –22018 polymorphisms and the incidence of hypolactasia is well known.

A decrease in lactase activity, progressing with age (LNP), is generated by genotypes -13910CC and -22018GG, while lifelong high lactase activity (LP) is produced by the presence of -13910CT and -13910TT as well as -22018GA and -22018AA genotypes. Recently, the role of DNA methylation in LNP/LP has also been reported [6].

Age gradual decrease in lactase activity can be associated to increasing methylation. Research suggests that LNP haplotypes containing the –13910*C allele accumulates modified cytosines that silence the regulatory elements in *LCT* rather than the haplotype containing the –13910*T allele. Age specific down-regulation and inter-individual variation of lactase activity in different populations may explain the epigenetic aging. Therefore, the epigenetic clock to regulate lactase expression is established by individual genetic background. Other genetic polymorphisms are also significant in other ethnic groups [4]. C/T –13914 (rs773131166) has been identified in some East Europeans [7], G/C –13907 (rs41525747) and A/C –13915 (rs41380347) in Middle East populations [8, 9], A/G –13913 (rs41456145) and C/G –14010 (rs145946881) are present in African populations and G/A –13908 (rs4988236) in Far East Asians [10]. These results suggest that LP alleles have emerged independently in several geographic/ethnic groups, a phenomenon most likely driven by recent positive selection due to the amount of dairy milk products used as food supply, as well as demographic factors (for example, migration) [11].

In countries in which the population is a mix of different ethnic groups, a method capable of testing all the possible SNP variations in one single reaction could be standardized and used, so that there is no need to tailor the investigation on patients’ origin.

To date, several diagnostic procedures are available to test lactose intolerance. The most common is Lactose breath test (LBT) that is a specific application of breath testing based on gaseous production from intestinal microflora [12]. LBT is highly specific and it has been considered the golden standard for the diagnosis of lactose intolerance; it however requires the patients to spend at least 4 h in the hospital; moreover, patients have to prepare themselves along the previous days. Genetic testing is a standard technique for the diagnosis of primary adult lactase persistence; it is a quick test, based on DNA extraction, usually from an EDTA blood sample or buccal swab [13], followed by real-time PCR protocol for the detection of a single SNP per reaction and Sanger sequencing, that is the most used method [14–17]. Other approaches have recently been developed, based on different techniques, like pyrosequencing [18], melting curve analysis [19], or loop-mediated isothermal amplification and melting curve analysis [20]. All these methods allow the simultaneous detection of several variants in the same reaction, although with different efficiencies. To recognize a secondary lactase deficiency can be crucial the contemporary administration of LBT and genetic testing [2, 21].

Secondary lactase deficiency is the consequence of many conditions leading to either a reduction of absorptive capacity or down-regulation of lactase expression in the small intestine, such as severe malnutrition, mucosal damage due to celiac disease and Inflammatory bowel diseases (IBD), Small bowel bacterial overgrowth (SIBO), etc. [22]. A secondary lactase deficiency is often reversible, when the cause is removed.

The present research was aimed to develop a novel genetic method to test lactase persistence more efficiently than the usual above-mentioned real-time PCR approach. This technique is based on the identification of SNPs with a single nucleotide primer extension method based on the principles of Sanger sequencing and capillary electrophoresis.

In this study, we selected eight different SNPs that are associated with lactase persistence [7–10] from Caucasian, Arabian Bedouins, sub-Saharian Africans and Asian populations (see Table 1) and are often very close to each other (some of them are adjacent in the genomic sequence). We set up an approach to detect all the eight different SNPs at the same time on the same sample.

**Table 1:** Shows the SNPs selected for this study, their variants and the geographic area in which they were originally identified

SNP	Variant	Population	SNP primer	TM	Name
rs41525747	G/C (− 13907)	Middle East	(GACT)_3_-AGGAGAGTTCCTTTGAGGCCA	55	SNPMDM1
rs4988236	G/A (− 13908)	Far East	(GACT)_5_-GGAGAGTTCCTTTGAGGCCAG	55	SNPMDM2
rs4988235	G/A (− 13910)	Europe	(GACT)_5_-GAGTTCCTTTGAGGCCAGGG	54	SNPMDM3
rs41456145	A/G (− 13913)	Africa	(GACT)_s_-CCTTTGAGGCCAGGGGCT	54.5	SNPMDM4
rs773131166	C/T (− 13914)	East Europe	(GACT)_9_-CCTTTGAGGCCAGGGGCTA	54.9	SNPMDM5
rs41380347	A/C (− 13915)	Middle East	(GACT)_11_-CTTTGAGGCCAGGGGCTAC	53.8	SNPMDM6
rs145946881	C/G (− 14010)	Sub Sahara Africa	(GACT)_9_-GGTATTAAATGGTAACTTACGTCTTTATG	53.7	SNPMDM7
rs182549	C/T (− 22018)	Europe	(GACT)_13_-ACAAAGGTGTGAGCCACCG	53.2	SNPMDM8

The column “SNP primer” contains the sequence designed for each SNP, followed by the Melting temperature (TM) of the region of homology with the chromosome and the primer name

## Materials and methods

The study was approved by the Ethics committee of University of Campania Luigi Vanvitelli. All enrolled subjects provided informed consent to participate.

Patients undergoing a Lactose breath test (LBT) for suspected lactose intolerance, over a twelve months period, were examined from Gastroenterology Unit of Azienda Policlinico Università della Campania L. Vanvitelli. 125 compliant subjects were enrolled, 75 females and 50 males mean age ± SD: 40.3 ± 14.8.

4 mL of peripheral whole blood was collected to perform genetic testing to the enrolled patients. Exclusion criteria were: age < 18 and > 75 years, major pathological and/or metabolic diseases and pregnancy.

### DNA extraction

Genomic DNA was extracted from EDTA anticoagulated blood with the QIAmp Mini Spin Column (QIAGEN, Hilden, Germany), following manufacturer’s protocol.

Briefly, 20 µl of protease-k and 200 µl of lysis buffer were added to 200 µl of sample (whole blood). The tube was vortexed for 15 s and incubated at 56 °C for 10 min, followed by centrifugation at 5000 rpm for 1 min. 200 µl of Ethanol were added and mixed for 15 s. After short centrifugation, the content of each tube was pipetted into the corresponding microfilter, and then centrifuged at 8000 rpm for 1 min. The flow-through was discarded and the micro filter washed with 500 µl of washing solution A, then with 500 µl of solution B. Genomic DNA was eluted in 100 μl of milliQ H_2_O.

Quantity and quality of the samples were measured with the Nanodrop LITE spectrophotometer (Thermo Scientific).

### DNA amplification and SNP analysis

Two primer pairs were designed to amplify the regions of interest in the lactase enhancer: 

MDM12F- ACTACTCCCCTTTTACCTCGTT, MDM12R- TCTGTTTATCTCTGCTCTCATCAT amplify a 400 bp region centered on the −  13910 position (rs4988235), and MDM22F- AGCTGGGACCACAAGCAC, MDM21R–CATTATCAGCCAACATCAAAGC amplify a 250 bp region surrounding the − 22018 position (rs182549).

The PCR reaction was conducted in a final volume of 25 μl with 0.5 μM of each primer and 1 unit of Taq Expand High Fidelity PCR System (Roche); the PCR protocol was 95 °C 3ʹ, then 35 cycles of 95 °C 30″, AT 30″, 72 °C 45″, followed by a final elongation step at 72 °C for 5ʹ. Annealing temperature was 56 °C for the MDM12F/12R primer pair and 58 °C for MDM22F/MDM21R primer pair; 0.5% DMSO were added in the MDM22F/MDM21R reactions.

For each sample, 5 μl of each PCR product were mixed and purified with ExoSap (Thermo Fisher), following manufacturer’s protocol. The DNA concentration for each PCR product was close to 10 ng/μl.

The following primers were designed for SNP detection (Table 1):

A GACT repeat was added to each primer to obtain the desired length. SNPs reaction was conducted with the SNaPshot Multiplex Kit (Thermo Fisher) following manufacturer’s protocol, with a final concentration of 0.2 μM for each primer (0.4 μM for the primers SNPMDM4 and SNPMDM5). Reactions were treated with Calf Intestinal Alkaline Phosphatase (CIAP, Thermo Fisher). 2 μl of each reaction was mixed with 0.2 μl GeneScan™ 120 LIZ™ dye Size Standard (Applied Biosystems), then loaded onto the 3730 DNA Analyzer (Thermo Fisher) following manufacturer’s instructions. Results were analysed with the GeneMapper 5 software (Thermo Fisher).

### DNA sequence analysis

Sequencing of the amplicons was performed using the BigDye Terminator v3.1 Cycle Sequencing Kit (Applied Biosystems), with the same primers described previously, in a 3730 DNA Analyzer (Thermo Fisher). The sequences were matched using BLAST (https://blast.ncbi.nlm.nih.gov/Blast.cgi).

## Results

**Fig. 1:**
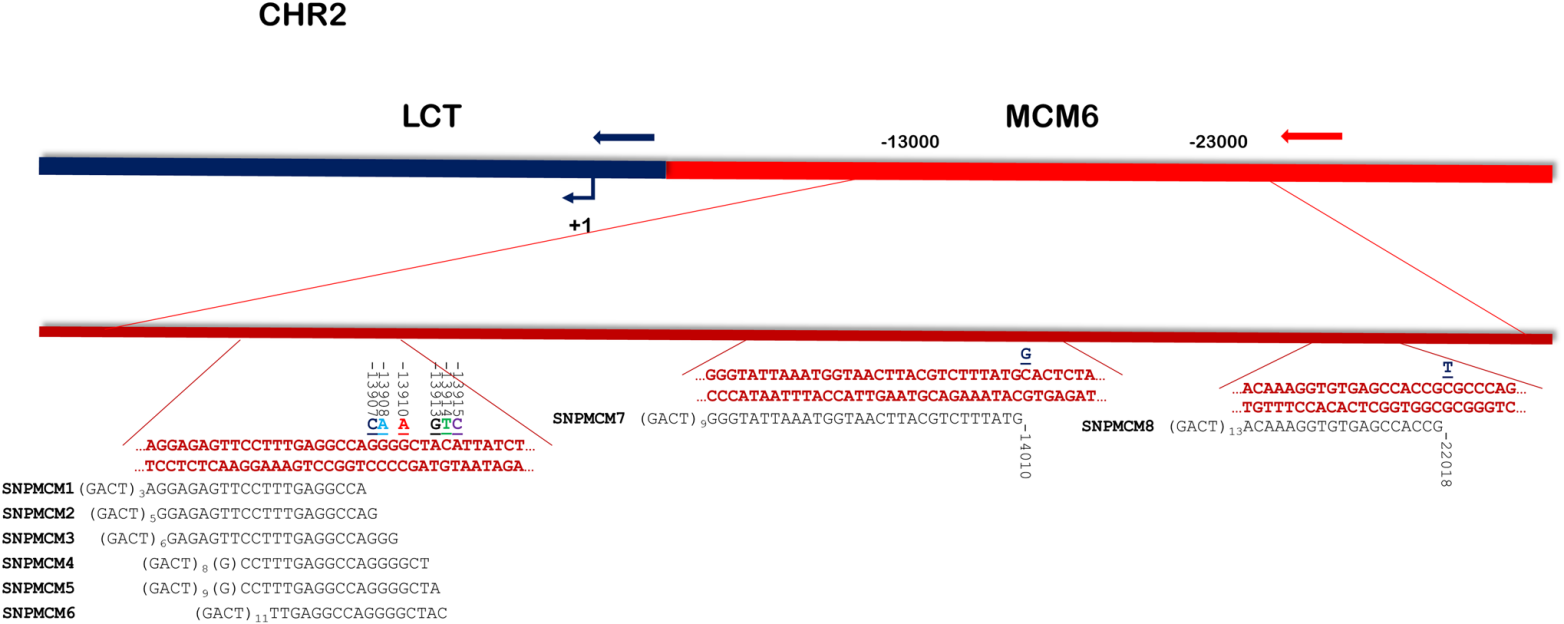
SNPs associated with lactase persistence. The double strand sequences surrounding the investigated SNPs are in red, the variants are indicated in different colors above the sequence. For each SNP, the SNP primer is aligned with the chromosomal sequence. (Color figure online)

Lactose intolerance was investigated in 125 individuals with a SNP analysis with the goal of developing a more reliable genetic test to evaluate lactase persistence. All the selected individuals were originally from Southern Italy and, due to the domination history of this region, we could not exclude a priori the presence of SNPs of different ethnic origin. The locus region of the LCT gene was analysed and, with the goal of obtaining a widely relevant genetic test, eight SNP previously reported to be associated with lactase persistence in different populations were identified (Fig. 1).

**Fig. 2:**
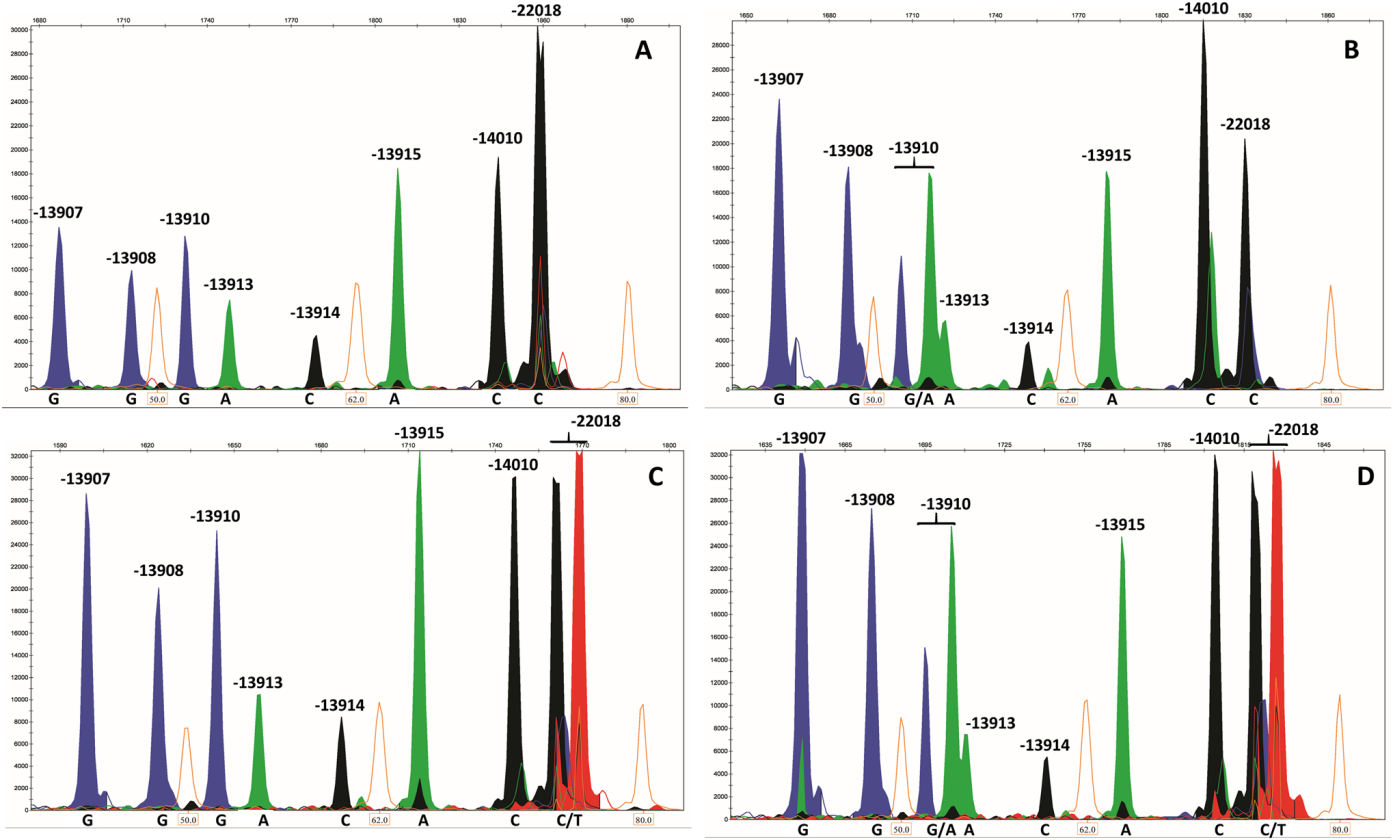
Examples of the eight SNPs analysis on four different individuals. Individual **A** WT; individual **B** Heterozygous in position − 13910; individual **C** heterozygous in position − 22018; individual **D** heterozygous in both − 13910 and − 22018 SNPs. Blue solid peaks represent Gs, green solid peaks represent As, black solid peaks are Cs and red solid peaks are Ts. Orange empty peaks, Molecular Weight Marker. (Color figure online)

A primer for each of them was designed, with a pairing region of 18–29 nucleotides preceded by a neutral GACT repetition, so that each final primer differed in length from the others of at least five nucleotides (Table 1), ranging from 36 to 71 bases in length. We tested all the primers in individual reactions with 2 μl of each purified PCR product to assess their performance before the setup of the multiplex assay with the SNaPshot multiplex kit (ThermoFisher) on the 3730 DNA Analyzer (ThermoFisher). We had to slightly increase the DNA amount for the multiplex reaction (5 μl of each PCR product) and, after testing different primer concentrations, we decided to double the amount of SNPMDM4 and SNPMDM5. We then performed a SNP analysis on all the 125 samples. With this approach we observed no variation for the positions − 13907, − 13908, − 13913, − 13914, − 13915 and − 14010, as expected in a caucasic population. Six subjects showed a C/T − 13910 heterozygous variation, seven were heterozygous C/T in position − 22018; 19 individuals were heterozygous in both positions (see Fig. 2 for representative results). We didn’t observe any homozygous variation in − 13910 nor in − 22018 SNPs (Table 2).

**Table 2:** Shows the distribution of the genotypes observed for SNPs − 13910 and − 22018 in our study

− 22018	− 13910		
	GG	GA	AA
CC	93 (74.4%)	6 (4.8%)	0 (0%)
CT	7 (5.6%)	19 (15.2%)	0 (0%)
TT	0 (0%)	0 (0%)	0 (0%)

**Fig. 3:**
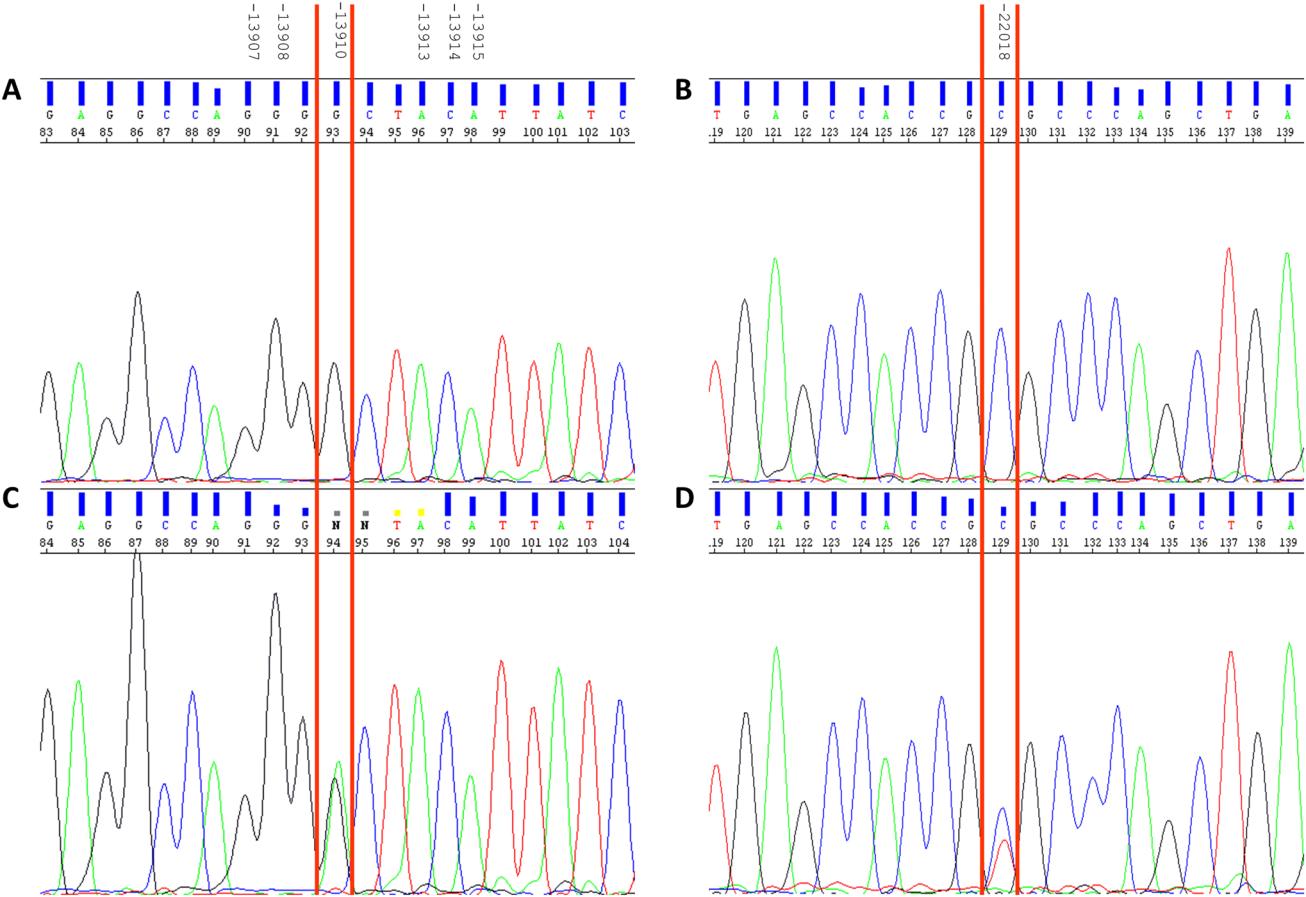
Two examples of applied Sanger sequence are shown. Sequences in **A** and **B** refer to the WT individual **A** of Fig. 2; **C** and **D** are the sequences of the double heterozygous individual **D** of Fig. 2. Red lines evidence the SNPs found heterozygous in individual **D**, resulted in two overlapped peaks, The other SNPs, indicated above, did not show variations in any individual, as the − 14031 SNP, not shown in this figure. (Color figure online)

In order to demonstrate the strength of our approach we decided to Sanger sequencing all the samples, to confirm the SNaPshot results in each case (Fig. 3). All the genotypes detected were confirmed by sequencing.

## Discussion

Lactose intolerance is widely recognized as one of the most common food adverse reactions. Although the hydrogen breath test is considered a golden standard to diagnose lactose intolerance, it poses some disadvantages, the most important being that it is not possible to distinguish between a primary or a secondary intolerance with this approach. The genetic basis of Lactase persistence (LP) or Non-persistance (LNP) is now well known, so genetic testing is becoming more and more used to obtain a better diagnosis of lactose intolerance and its clinical value increases with age [23], being maximal in adulthood. A bias in this approach is the presence of different SNPs in the enhancer region of the lactase gene, strictly associated with the ethnicity of individuals [24]. The usual genetic test, the detection by a real time PCR approach, commonly investigates one SNP per reaction, implies the knowledge of the ethnicity of each individual. To overcome this limitation, we developed a SNP assay to investigate different genetic variants associated with lactase persistence at the same time in the same reaction. We tested 125 individuals and confirmed all the obtained results by Sanger sequencing, finding 100% congruence.

In the modern, globalized world, more and more countries have their population composed of people from different geographical areas. Studies of the genetic predisposition to lactase persistence showed a strong association between the ethnicity and a given polymorphism. Our work demonstrated that the SNP approach, while as informative as the Real Time PCR technique, has the advantage of a single standardized reaction applicable to all known lactase polymorphisms independently of the ethnicity of the patients.

Lactase persistent phenotype is incredibly variable globally. In Europe, a North–South distribution can be detected; in fact, 90% of adults can digest lactose in Scandinavian countries, however, lactose tolerant adults are less than 50% in Mediterranean. In Italy, frequencies of 62, 3% non-persistent genotypes while in southern Italy 67, 1% have been recorded [25, 26].

The observed percentage of non-persistent genotypes in this study is higher (i.e.: 74, 4%) than the above reported values, possibly because patients were already suspected to have lactose intolerance and enrolled during a lactose intolerance screening.

LCT genotyping could replace or complement hydrogen breath testing, which is laborious for both patients and physicians, and can be affected by age, and other minor causes as celiac disease [27]. Moreover, beside venous blood used here, LCT genotyping could be performed using buccal swab procedure that is suitable for large population-based genetic studies [28] and saliva, which has been shown to be an excellent source of human genomic DNA [29]. Checking saliva we can obtain a less invasive method compared to blood or cotton swab samples.

The traditional Real Time PCR approach allows the detection of a single SNP per reaction, but it is increasingly interesting the development of techniques that permit the simultaneous detection of different SNPs in one reaction, especially in countries target of human migratory flows. Other methods have already been developed, all having advantages and disadvantages. Pyrosequencing [18] is efficient, but it is useful only for SNPs that are within a short distance from each other, it would not be possible to analyse, for example, positions − 13910 and − 22018 in the same reaction. Melting curve analysis [19] and loop-mediated isothermal amplification and melting curve analysis [20] are easier than sequencing or SNPs detection, but they often do not resolve some different genotypes, and they both request the use of standard sequences to identify the correct SNPs in genomic DNAs [19, 20].

We think that the SNP Assay that has been validated in this study represents a valuable tool for routine diagnosis, especially if applied to saliva genomic DNA, and even if it requires DNA extraction and an automated sequencer.

In fact the combination of eight different SNP allows the accurate molecular typing of multiple known LCT variants and overcomes diagnostic pitfalls reported for other methodologies. It is worth to underline that the potential of this approach could be wider because more than eight SNPs could still be analysed in each sample setting up a single reaction tube per patient.
